# Supplementary material for: β-Hydroxybutyrate Increases Exercise Capacity Associated with Changes in Mitochondrial Function in Skeletal Muscle
Source: Nutrients. 2020 Jun 29;12(7):1930. doi: 10.3390/nu12071930 (PMC7400376; doi:10.3390/nu12071930)
Supplement: Supplementary file 1 [file nutrients-12-01930-s001.zip › Supplementary Figure 1.pdf]

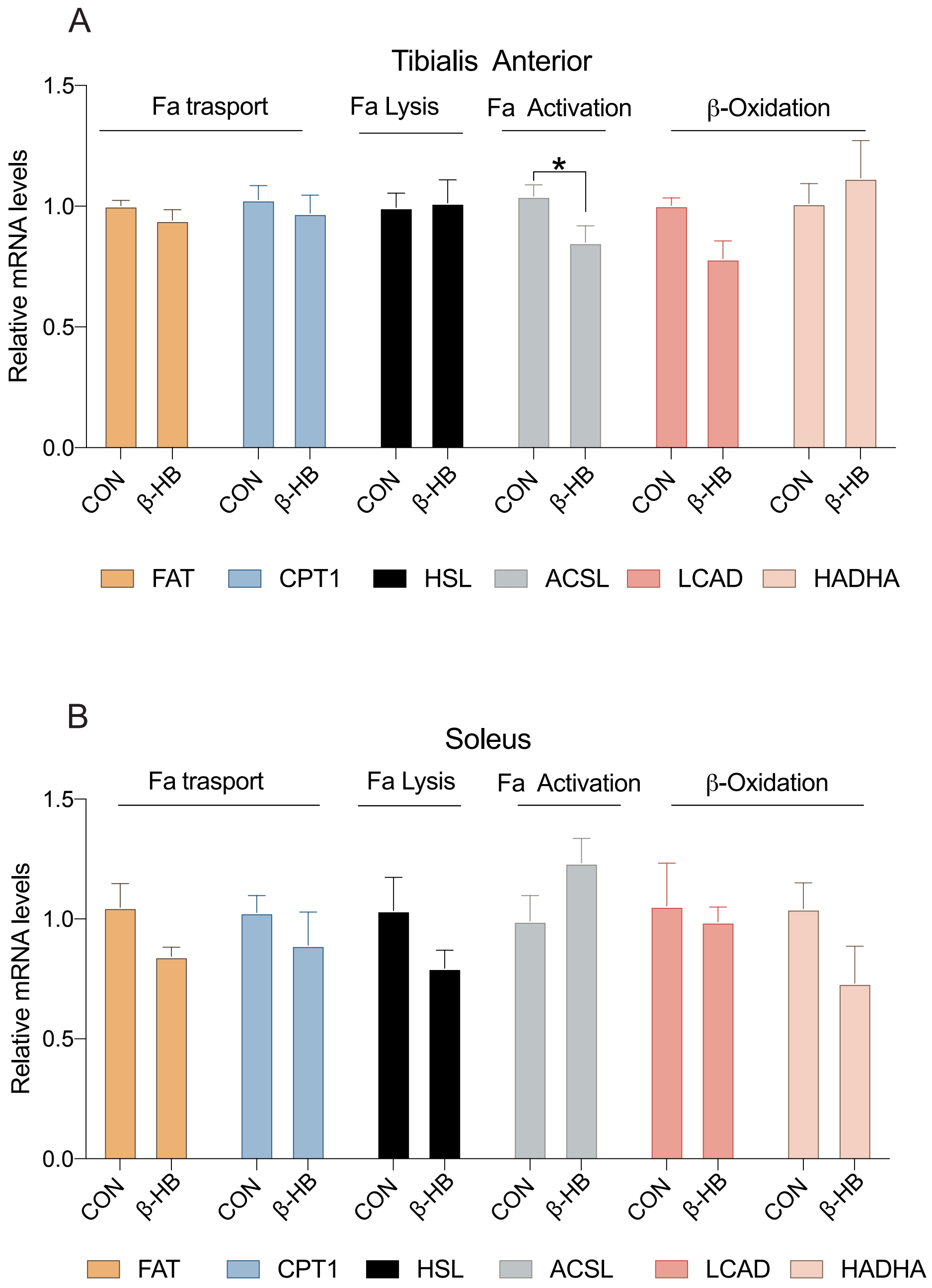

Supplementary Figure 1. Short term b-hydroxybutyrate supplementation effects on Fatty acid lysis, transport, and b-Oxidation mRNA levels. Tibialis anterior: (A) Hormone Sensitive Lipase (HSL), Fatty acid Transport Protein (FAT), Carnitine palmitoyl Transferase 1 (CPT1), Long-chain Acyl-CoA Dehydrogenase (LCAD), Long Chain Acyl-CoA Synthetase (ACSL)and Hydroxyacyl-CoA Dehydrogenase Subunit alpha (HADHA). Soleus: (B) Hormone Sensitive Lipase (HSL), Fatty acid Transport Protein (FAT), Carnitine palmitoyl Transferase 1 (CPT1), Long Chain Acyl-CoA Synthetase (ACSL)- Very long-chain Acyl-CoA dehydrogenase (VLCAD) and Hydroxyacyl-CoA Dehydrogenase Subunit alpha (HADHA). n=4 per group *p<0.05, Unpaired t-Test. Values expressed as mean ± SEM.
