# Supplementary material for: β-Hydroxybutyrate Increases Exercise Capacity Associated with Changes in Mitochondrial Function in Skeletal Muscle
Source: Nutrients. 2020 Jun 29;12(7):1930. doi: 10.3390/nu12071930 (PMC7400376; doi:10.3390/nu12071930)
Supplement: Supplementary file 1 [file nutrients-12-01930-s001.zip › Supplementary Table 1.docx]

**Supplementary Table 1. List of Primers**

| Gene | Forward primer | Reverse Primer |
| --- | --- | --- |
| Fatty Acid Transport Protein (FAT) | 5’ TGACAGTGCCACCAACAAGAA 3´ | 5’ GCGCTATCGCCCTTTCG 3’ |
| Hormone Sensitive Lipase (HSL) | 5’ ACTGAGATTGAGGTGCTGTC 3’ | AGGTGAGATGGTAACTGTGAG 3’ |
| Long Chain Acyl-CoA Synthetase (ACSL) | 5’ CGCACCCTTCCAACCAACAC 3’ | 5’ TCGTCGTAGTACACCAAGAGC 3’ |
| Carnitine Palmitoyl Transferase 1(CPT1) | 5’ GTGCAAGCAGCCCGTCTAG 3’ | 5’ TTGCGGCGATACATGATCAT 3’ |
| Long chain specific acyl CoA dehydrogenase (LCDA) | 5’ AAGGATTTATTAAGGGCAAGAAGC 3’ | 5’ GGAAGCGGAGGCGGAGTC 3’ |
| Long-chain-3 hydroxyacyl-CoA dehydrogenase(HADHA) | 5’ GGCCTGTCACTGGTAGAACT 3´ | 5’ GGCCTGTCACTGGTAGAACT 3´ |
